# Theoretical analysis of hot electron dynamics in nanorods

**DOI:** 10.1038/srep12140

**Published:** 2015-07-23

**Authors:** Chathurangi S. Kumarasinghe, Malin Premaratne, Qiaoliang Bao, Govind P. Agrawal

**Affiliations:** 1 Monash Advanced Computing and Simulation Laboratory (AχL), Department of Electrical and Computer Systems Engineering, Monash University, Clayton, Victoria 3800, Australia; 2 Department of Materials Science and Engineering, Monash University, Clayton, Victoria 3800, Australia; 3 Institute of Functional Nano and Soft Materials (FUNSOM), Jiangsu Key Laboratory for Carbon-Based Functional Materials and Devices, and Collaborative Innovation Center of Suzhou Nano Science and Technology, Soochow University, Suzhou 215123, P. R. China; 4 The Institute of Optics, University of Rochester, Rochester, New York 14627, USA

## Abstract

Localised surface plasmons create a non-equilibrium high-energy electron gas in nanostructures that can be injected into other media in energy harvesting applications. Here, we derive the rate of this localised-surface-plasmon mediated generation of hot electrons in nanorods and the rate of injecting them into other media by considering quantum mechanical motion of the electron gas. Specifically, we use the single-electron wave function of a particle in a cylindrical potential well and the electric field enhancement factor of an elongated ellipsoid to derive the energy distribution of electrons after plasmon excitation. We compare the performance of nanorods with equivolume nanoparticles of other shapes such as nanospheres and nanopallets and report that nanorods exhibit significantly better performance over a broad spectrum. We present a comprehensive theoretical analysis of how different parameters contribute to efficiency of hot-electron harvesting in nanorods and reveal that increasing the aspect ratio can increase the hot-electron generation and injection, but the volume shows an inverse dependency when efficiency per unit volume is considered. Further, the electron thermalisation time shows much less influence on the injection rate. Our derivations and results provide the much needed theoretical insight for optimization of hot-electron harvesting process in highly adaptable metallic nanorods.

Surface plasmons in nanostructures that can concentrate and guide energy on a nano scale are generated by coherent oscillations of free electrons coupled to an incident electromagnetic field. The nano-scale confinement of surface plasmons not only creates highly energetic electrons through nonradiative decay but these electrons can also be injected into other neighboring materials for aiding applications such as photovoltiacs^1^,^2^, photocatalysis^3^,^4^,^5^,^6^,^7^, photodetection^8^,^9^,^10^, nano-scale imaging^11^, photo-induced phase transitions^12^ and doping of other materials^13^. The efficiency of this injection process depends highly on the shape of the nanostructure employed^14^.

When harvesting solar energy, semiconductor materials are typically used to absorb solar photons and to generate electron-hole pairs, causing current flow or fueling catalytic activity of chemical reactions. To generate electron-hole pairs inside a semiconductor, photon energy should be higher than the semiconductor bandgap energy, a requirement that critically limits the efficiency of such a process. When a metal nanostructure is in contact with a semiconductor, even the photons that have energy lower than the bandgap can generate energetic electrons inside the nanostructure through a surface plasmon resonance, which can then cross the metal—semiconductor Schottky barrier and enter the conduction band of the semiconductor. Since this barrier energy is typically much lower than the associated semiconductor bandgap, electrons could jump over more easily than direct electron excitations in the semiconductor^15^. Further, the ability to tune the plasmon resonance of nanoparticles by changing their composition, shape or dimensions^16^,^17^,^18^ gives one the flexibility for tailoring the operating frequency of such hybrid structures, and thus the potential of harvesting photons over the entire solar spectrum. In addition to photovoltaic applications, such electrons and holes can participate in chemical reactions on the metal or the semiconductor surface^3^,^4^,^5^,^6^,^7^.

The preceding scheme has already been exploited to realise an efficient solar water-splitting system whose operation is based on gold nanoparticles that generate hot electrons through surface plasmons^3^. Many nanostructures of different shapes and material have been experimentally tested recently to improve the efficiency of such a hot-electron transfer process, and plasmon to hot electron conversion efficiency of about 30% has been achieved by concentrating propagating surface plasmons into a tapered conical metal tip^11^. The use of localised surface plasmons provided only a 2.75% quantum yield using a hybrid structure of CdS nanorods with a gold sphere at one end^19^. In another experiment, hot-electron transfer from a plasmonic nanostructure to a nearby graphene sheet yielded a quantum efficiency of 10%^20^. Experimentally reported efficiencies depend on the height of the Schottky barrier, which depends strongly on many details of the metal—semiconductor interface along with the work functions of the two materials.

Surface roughness of a metal—semiconductor interface also plays an important role in the injection efficiency^21^. When the interface is smooth, owing to linear momentum conservation requirements, a high-energy electron can cross over to the conduction band of the semiconductor, provided the component of its momentum normal to the interface is higher than the energy of the Schottky barrier. This rule is not strictly followed if the surface is rough, resulting in higher injection efficiencies.

The non-equilibrium, high-energy electrons created by surface plasmons relax subsequently by emitting photons, distributing their energy among other electrons (in several hundred femtoseconds), and transferring energy to the lattice within a duration of few picoseconds^22^,^23^,^24^. Clearly, the injection of hot electrons over an energy barrier should happen before electrons lose energy by these relaxation processes. When electrons are injected to the semiconductor, the metal making up the Schottky barrier will have a deficit of electrons. An efficient electron donor mechanism should be available to maintain the injection rate^3^,^25^ because any backward transfer of electrons from the semiconductor to the metal nanoparticle would reduce the efficiency of the process.

The size of the nanostructure is among the most important parameters when it comes to hot-electron injection^26^,^27^. In small nanoparticles, the dominant mechanism of plasmon decay is through nonradiative energy transfer to the electron gas rather than radiative decay^28^. Further, the injection efficiency is high when distance the hot electrons have to travel to reach the metal-semiconductor boundary is relatively small so that they have less opportunity to lose energy through different relaxation processes along the migration path. For this reason, smaller nanoparticles can be expected to have higher efficiencies than their larger counterparts. It has been shown theoretically for a nanopallet that electron injection efficiencies reduce with increasing pallet width^26^.

Electric field enhancement inside a nanoparticle significantly influences the generation of hot electrons, and this enhancement depends mainly on the nanoparticle shape. Also, the direction of the applied electric field relative to the semiconductor metal interface plays a crucial role in setting the fraction of hot electrons that have the correct momentum orientation for injection over the Schottky barrier. The electric field enhancement factor is the highest inside nanorods or ellipsoidal nanostructures supporting a longitudinal palsmon resonance^14^, compared with other basic shapes such as nanospheres, nanocubes or nanopallets. Therefore, a nanorod is an important shape for hot-electron based applications. A solar water splitting system based on relatively large gold nanorods (diameter 90 nm and length >150 nm) capped with TiO_2_ has shown promising results experimentally^4^. But for relatively small nanorods for which the efficiency is expected to be higher, a proper theoretical analysis is lacking in the literature even though it has been done for other shapes such as spheres, shells, cubes and pallets.

Typically, Fowler theory is used to explain photoemission from a metal to a semiconductor^29^. This theory assumes an isotropic distribution of electron momentum orientations inside the metal, which is not the case for small nanoparticles with dimensions less than the electron mean free path^30^. The electron mean free path for silver and gold has reported values of 50 nm and 40 nm, respectively, at energies close to the Fermi energy^31^,^32^. Further, Fowler theory requires a structure-specific coefficient, which needs to be measured experimentally. Quantum-mechanical theories have been developed to analyse the interesting phenomena of hot electron transfer that have incorporated the shape and momentum conservation. These have been used to find theoretically the device-specific coefficients of Fowler theory^26^,^27^.

An alternative is to use an ab-initio approach based on density functional theory in which detailed shape and size dependant potential information inside the nanoparticle is taken into account by treating the whole system as a quantum-mechanical many-body problem before calculating the rates of hot-electron generation and injection. However, a many-body approach is likely to be limited to nanoparticle sizes of less than 5 nm owing to its excessively high computational costs. Moreover, such a full-blown numerical analysis will lead to loss of insight that is very valuable to engineer practical devices and systems. For this reason, we have adopted in this work a much simpler single-electron model, which assumes a non-interacting electron gas confined under a uniform background potential. In this model the single-electron wave function represents all properties of a nanoparticle. This method is suitable for situations where electrons behave as a free-electron gas under a uniform background potential. Since electrons in metals behave in this fashion, our method is suitable for modeling quantum mechanical properties of metals. We use it to obtain numerically the size and shape dependant hot electron injection rate for a nanorod with dimensions less than electron’s mean free path. This model has been successfully adopted for other geometries such as nanopallets and nanocubes^14^,^26^, and its results have been successfully matched with detailed DFT calculations for nanospheres^27^.

In this work, we derive the generation and injection rates of hot electrons inside an optically excited metal nanorod in contact with a semiconductor and use them to investigate the dependence of these two rates on the thermalisation rate of electrons and on the volume and aspect ratio of the nanorod. We also compare the injection rate of nanorods with nanospheres and nanopallets to show that nanorod is the most desirable shape for hot-electron harvesting over a broad spectral range.

## Theoretical Formulation and Analysis

**Figure 1:**
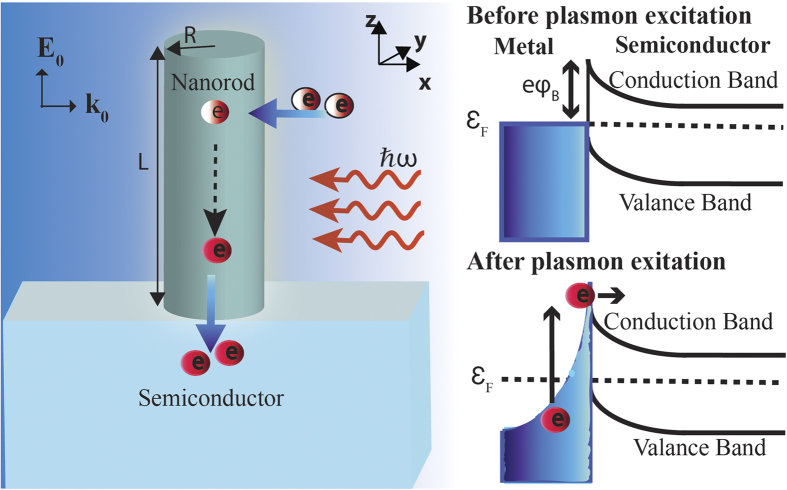
Schematic showing generation of hot electrons inside an optically excited metal nanorod and their injection from naorod to the semiconductor over the Schottky barrier formed at the metal—semiconductor interface. The incident light is propagating in the direction of the wave vector **k**_0_ with its electric field **E**_0_ oriented along the length of nanorod.

As shown in Fig. 1, we consider a metallic nanorod of length *L* and radius *R* in contact with a semiconductor at its bottom side. Light of frequency *ω* is incident on the nanorod from one side with its electric field **E**_0_ oriented along the length of nanorod. Let *i* and *f* represent the initial and final states of an electron inside this nanorod before and after the external perturbation. In the random phase approximation, the generation rate 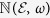 of hot electrons of energy 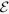 can be calculated using^26^,^27^,


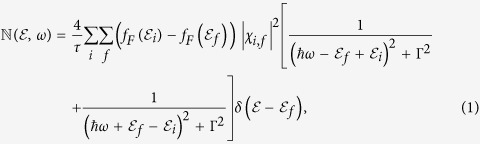


where a factor of 2 is included to account for the electron spin and


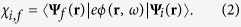


The variables *ħ*, *e* and *ϕ*(**r**, *ω*) are reduced plank constant, electron charge and internal potential at frequency *ω*, respectively, with **r** denoting the position vector and 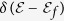 denoting the Dirac delta function. The variables Ψ_*k*_(**r**) and 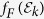 indicate the wavefunction and the Fermi distribution associated with an electron’s state of energy 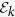, respectively. Γ = 2*πħ*/*τ* is the rate of electron thermalisation in the metal, where *τ* is the thermalisation time. In order to apply this formalism, we first need to find the wave function, energy levels and the potential of an electron inside the nanorod.

### Wave function of an electron inside a nanorod

Assuming that conduction electrons in the metal are free particles and the nanorod boundary is impenetrable to them, the Schrödinger equation for a single electron can be written in cylindrical coordinates as,


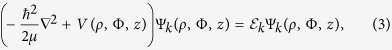


with the confining potential *V*(*ρ*, Φ, *z*) given by


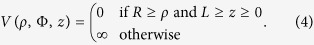


Owing to the cylindrical symmetry of the nanowire, the wave function Ψ_*k*_(**r**) can be separated in to orthonormal functions, *ψ*_*n*,*m*_(*ρ*), *ψ*_*m*_(Φ) and *ψ*_*l*_(*z*) that represent the radial, azimuthal and longitudinal components of the wave function respectively:


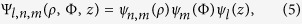


where the integers *n* ≥ 0, |*m*| ≥ 0 and *l* ≥ 0 are quantum numbers corresponding to a particular electron state *k*. By solving the eigenvalue problem after substituting Eqs. (4) and (5) in Eq. (3), we find the longitudinal component of the wave function needed for further calculations in the form


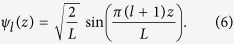


Energy of an electron in a cylindrically confined quantum well in a state indicated by quantum numbers *n*, *l* and *m* is found to be


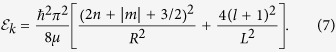


### Potential inside a nanorod

We assume that the electric field of incident radiation is linearly polarized along the longitudinal axis of the nanorod since longitudinal modes of electrons show higher extinction than the transverse modes^33^. As our focus is on subwavelength nanorods, we take the dimensions of the nanorod to be small compared to the wavelength of the incident radiation. Therefore, we can assume that the incident electric field is uniform and does not vary spatially inside the particle^34^. According to Maxwell’s equations, the electric field vector inside the particle, **E**(*ω*) can be related to the potential inside the particle as **E**(*ω*) = −**∇***ϕ*(*r*, *ω*). Although no exact analytical solution exists for the electric field **E**(*ω*) inside a nanorod, it is known that the plasmonic behaviour of a cylinder is quite close to an ellipsoid when *L* ≫ *R*^35^. For this reason, we approximate the nanorod as an ellipsoid and write the internal electric field in terms of the externally applied field **E**_0_(*ω*) as **E**(*ω*) = *γ*(*ω*)**E**_0_(*ω*), where the electric field enhancement factor *γ*(*ω*) depends strongly on the shape of the nanoparticle and its orientation with respect to the applied field. When the applied electric field is along the longitudinal axis of a nanorod, the internal electric field enhancement factor *γ*(*ω*) depends on the complex permittivity of the metal *ε*(*ω*) as^36^,


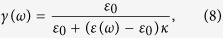


where *ε*_0_ is the permittivity of the external medium and


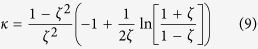


depends on the eccentricity of the nanorod given by *ζ* = (1 − 4*R*^2^/*L*^2^)^1/2^. It is clear from this equation that the field inside the nanorod is uniform and parallel to the external field. When *L* is comparable to or less than *R*, the electric field enhancement factor cannot be described by Eq. (8). In our analysis, for nanorods with aspect ratios <2, electric field inside the nanoparticle was calculated numerically and the elctric field enhancement factor was derived as^14^,


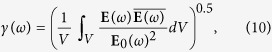


where *V* is the volume of the nanoparticle.

### Hot electron generation rate

The matrix element *χ*_*i*,*f*_ can be written in terms of the magnitude of the external electric field **E**_0_(*ω*) polarized along the 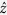 direction as,


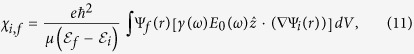


where 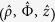 are the unit vectors of the cylindrical coordinate system and *μ* represents electron’s mass. In deriving this, we have used the quantum mechanical relationship between the position operator *r* and the momentum operator 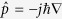 defined as^37^,


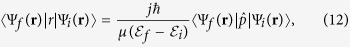


with *j* denoting unit imaginary number. Using the electron wave function from Eq. (5) we can write


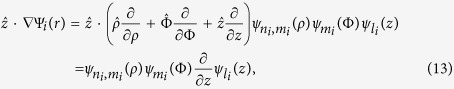


where quantum numbers *m*_*i*_, *n*_*i*_ and *l*_*i*_ indicate the state *i* and *m*_*f*_, *n*_*f*_ and *l*_*f*_ indicate the state *f*. The derivative of the longitudinal component of the wave function is easily calculated to be


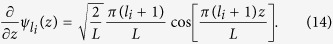


By substituting Eq. (13) and Eq. (14) in Eq. (11), the matrix element *χ*_*i,f*_ takes the form


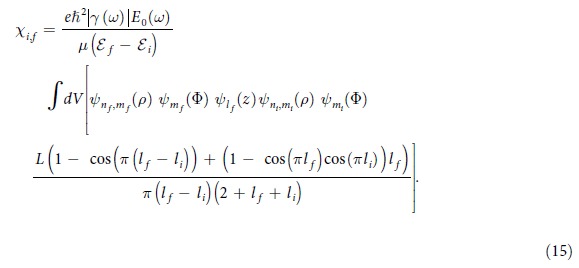


This equation shows that *χ*_*i*,*f*_  = 0 for non-odd values of *l*_*f*_  − *l*_*i*_. Therefore, after taking into account the orthonormal nature of the wave function, we can simplify *χ*_*i*,*f*_ for odd values of *l*_*f*_  − *l*_*i*_ as


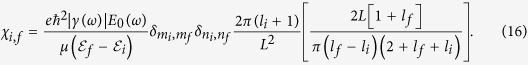


Because of the presence of 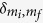 and 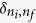 in Eq. (16) it is clear that *m*_*i*_ = *m*_*f*_ and *n*_*i*_ = *n*_*f*_ is required for *χ*_*i*,*f*_ to be non-zero. For this reason, we need to consider in our analysis those transitions that change only the longitudinal quantum number. Physically, this condition is imposed because the applied electric field is along the longitudinal axis of the nanorod and the internal electric field is in the same direction as the applied field. The energy difference between states *f* and *i* using Eq. (7) is found to be


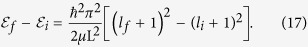


After substituting Eq. (17) in Eq. (16) we arrive at the expression,


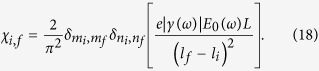


Using the preceding expression or the matrix element, the generation rate of hot electrons is found from Eq. (1) to be


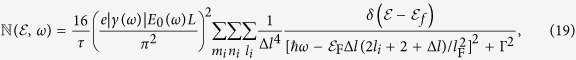


where Δ*l* = *l*_*f*_ − *l*_*i*_ with the condition Δ*l* = 1,3,5….... and 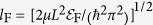. The limits of summation for quantum numbers *m*_*i*_, *n*_*i*_ and *l*_*i*_ can be found by noting that the electron will initially occupy a state below the Fermi level and during the transition it will move to an energy state above the Fermi level.

The electron injection rate 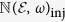 from the nanorod to the semiconductor in contact with it can be calculated from 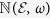 by considering only situations where the excited electrons have the longitudinal component of their energy greater than the sum of the Fermi energy and Schottky barrier height between the metal and the semiconductor (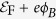). Same as before, they are assumed to occupy states below the Fermi level before excitation. These conditions can be conveniently written as


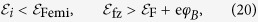


where 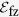 is the longitudinal component of the electron energy 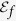.

**Figure 2:**
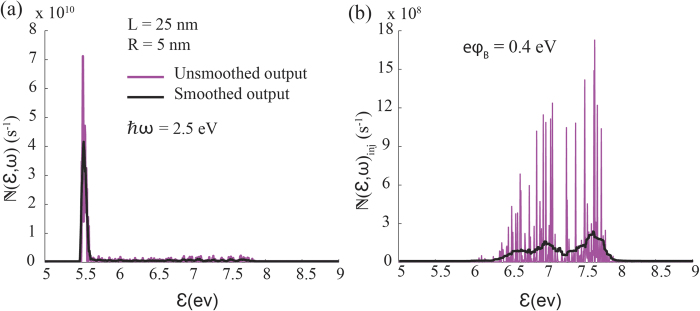
Energy distribution of (**a**) generated hot electrons and (**b**) injected hot electrons over a barrier of 0.4 eV at *ħω* = 2.5 *eV* for a nanorod of volume 1000 nm^3^ and aspect ratio 2.5. In all calculations, electron’s thermalisation time in silver is taken to be 350 fs. The smoothed output shown in Fig. 2a and Fig. 2b was calculated by taking the moving average with a low pass filter with filter coefficients equal to the reciprocal of the span to capture the general pattern.

Figure 2 shows the energy distribution of generated and injected electrons calculated from Eq. (19) for the particular case of Ag/TiO_2_ metal-semiconductor system in water. More specifically, photons of 2.5 eV energy are incident on the silver nanorod such that the electric field is polarized along the longitudinal axis of the nanorod. It can be seen that most generated electrons have energies in the vicinity of the Fermi level of the metal (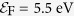), and therefore only a small fraction of of these electrons has sufficient energy and correct momentum orientation with respect to the metal-semiconductor interface to cross the Schottky barrier and to enter the semiconductor. Similar behaviour can be observed in other shapes such as nanospheres, nanoshells and nanopallets^26^,^27^.

### Hot-electron generation and injection rates as a function of frequency

By summing 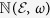 over all possible values of 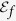 we obtain the generation rate of hot electrons, 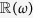, as a function of the frequency *ω* of the incident radiation:


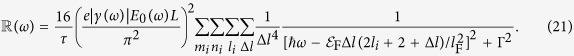


Electron injection rate as function of frequency can be obtained by imposing the conditions in Eq. (20) on 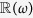. We derive upper and lower limits of *l*_*i*_, *mi* and *ni* from these conditions as,


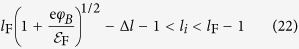



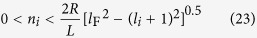



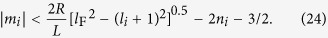


If we consider the sum over *m*_*i*_ and *n*_*i*_ in Eq. (21), it can be seen that it corresponds to a degeneracy factor that can be easily calculated using the preceding limits as


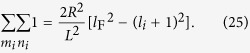


Using this result the injection rate of hot electrons as a function of incident radiation frequency is found to be


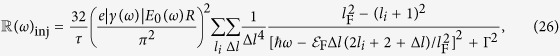


with the condition 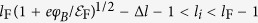. It can be seen from the preceding equation that the injection rate hot electrons is proportional to Δ*l*^−4^ and therefore it is desirable to excite electrons to energy levels that minimize the change in longitudinal quantum number Δ*l*. It can be argued that the spacing between energy levels becomes large when dimensions of nano particles are small, allowing electrons to reach higher energy levels with a relatively small change in the longitudinal quantum number.

**Figure 3:**
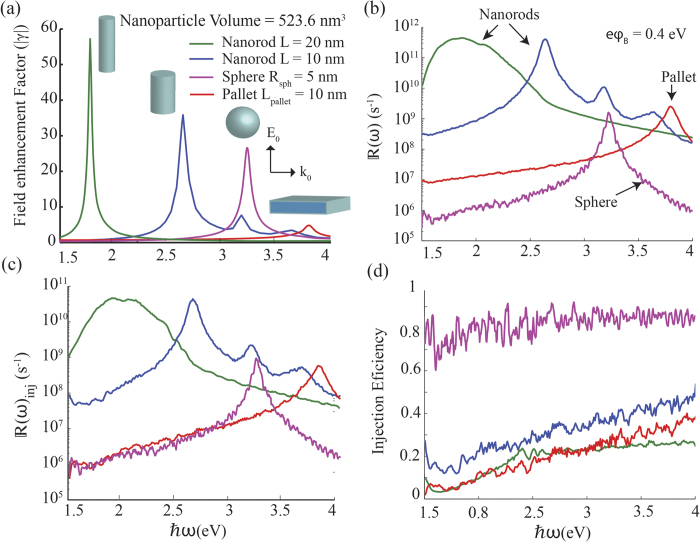
Comparison of (**a**) electric field enhancement factors, (**b**) electron generation rates, (**c**) electron injection rates and (**d**) injection efficiency for a nanosphere, a nanopallet, and a nanorod made of silver having the same volume. The Schottky barrier height is taken is 0.4 eV and electron thermalisation time in Ag is taken to be 350 fs for nanorod with L = 20 nm and 300 fs for all other cases. The incident light is propagating in the direction of the wave vector **k**_0_ with its electric field **E**_0_ oriented vertically. *R*_sph_ and *L*_pallet_ are radius of the sphere and the thickness of the pallet respectively.

Figure 3 compares the electric field enhancement factors and electron generation and injection rates and electron injection efficiency of a sphere, pallet, and two nanorods, all having the same volume. It is apparent from Fig. 3(a) that nanorods have a much stronger electric field enhancement factors (|*γ*(*ω*)|), which depends strongly on the aspect ratio, when compared to the other two geometries. Further, as shown in Fig. 3(b,c), nanorods can be tuned with their aspect ratio to obtain electron generation and injection rates that are several orders of magnitude higher than spheres or pallets of similar volume. The injection and generation rates of hot electrons are proportional to |*γ*(*ω*)|, and they also depend on details of the electron energy levels associated with the nanostructure. Both of these factors depend on the shape of the nanostructure, and collectively they contribute to efficient generation of high energy electrons for a particular nanorod. It can be seen from Fig. 3(d) that sphere has the highest injection efficiency which indicates that a significant amount of hot electrons generated have sufficient energy and momentum orientation to be injected into the surrounding medium. Here we have assumed for a sphere that any electron having sufficient energy and momentum in the radial direction can be injected. However, it can be clearly seen that the overall electron injection rate, which is the quantity of importance in energy harvesting applications is much higher in nanorods compared to spheres.

The non-Fermi electron distribution created in nanoparticles after the application of an electric field thermalize internally through electron-electron scattering leading to a hot Fermi distribution. This internal electron thermalization pracess takes place within several hundred femtoseconds^38^. The elctron thermalization time *τ* can be expected to reduce with the nanoparticle size when the size is reduced below the electron mean free path of the conduction electrons (≈50 nm for Ag), as a result of increased interaction between the surface and the electrons, assuming these interactions are inelastic. However, it has been observed experimentally that for large enough spherical noble metal particles (typically above 10 nm in diameter) the electron thermalization time is not strongly size dependant and it is comparable to the values measured in the bulk due to elastic nature of electron-surface interactions^38^,^39^,^40^.

Experimental measurements of *τ* in bulk metals can vary over a wide range depending on the factors such as the surface quality at the boundary of the metal, electron excitation power, and the lattice temperature^41^,^42^. For silver films, reported values of *τ* in literature vary from 50 fs to 1000 fs and generally taken as 350 fs in most calculations^23^,^40^,^41^. For particles with dimensions above 10 nm, we have used the bulk thermalization time in our calculations. For the case of 10 nm particles in Fig. 3 we have taken this value to be 300 fs^40^. The reduction of thermalization time is due to increased electron-electron interaction as a result of less efficient screening of the of Coulomb interactions close to the surface in small nano particles.

When considering the spectrum of solar energy reaching Earth’s surface, photons in the range of 1.77 eV (700 nm) to 3.10 eV (400 nm) corresponding to the visible region represent 42%–43% of the total energy received from the sun^43^,^44^,^45^. Another 50%–55% consists of photons in infrared region having energies below 1.77 eV (wavelength > 700 nm). Only 3%–5% have energies in the ultraviolet range with energies above 3.10 eV (wavelength < 400 nm). Therefore, we can confidently state that nanorods are suited for solar energy harvesting applications nanorods perform better than both pallets and spheres in solar energy harvesting applications since they show much higher injection rates in the range 1.5 eV to 3.10 eV where a high percentage of energetic photons are received.

**Figure 4:**
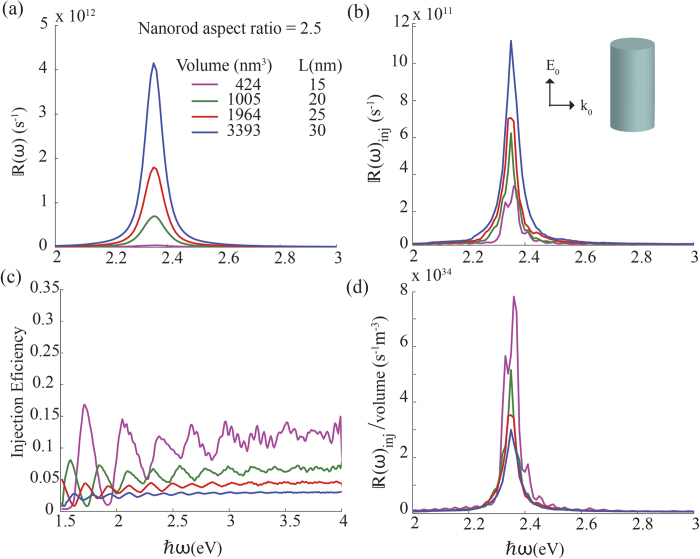
Comparison of hot-electron dynamics for silver nanorods with a constant aspect ratio of 2.5 but varying volume. Part (**a**) show the calculated generation rate of hot electrons and (**b**) shows their injection rates from the nanorod to a semiconductor over a Schottky barrier height 0.4 eV. Parts (**c**) and (**d**) show the electron injection efficiency and quantum efficiency of the nanorods, respectively. In all calculations electron thermalisation time is 350 fs and incident light is propagating in the direction of the wave vector *k*_0_ with its electric field oriented vertically.

Figure 4 shows the effects of varying the volume of a nanorod while keeping the aspect ratio constant. According to Eq. (8), the electric field enhancement factor of a nanorod only depends on its aspect ratio and not on its volume, i.e., |*γ*(*ω*)| is constant for all nanorods in Fig. 4. Since the number of conduction electrons increases with increasing volume, there is in an overall increase in hot-electron generation and injection rates, as seen in Fig. 4(a,b). At the same time, the injection efficiency shown in Fig. 4(c), calculated as 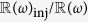, reduces as the the volume is increased. Even though higher volume nanorods generate a larger number of excited electrons, a high proportion of them have energies in the vicinity of the Fermi level, and therefore they lack sufficient energy to cross over the Schottky barrier. As a general trend, the injection efficiency per unit volume plotted in Fig. 4(d) is declining with an increase in the nanoparticle volume. Therefore, nanorods with a smaller volume show better performance when injection per unit volume is considered.

**Figure 5:**
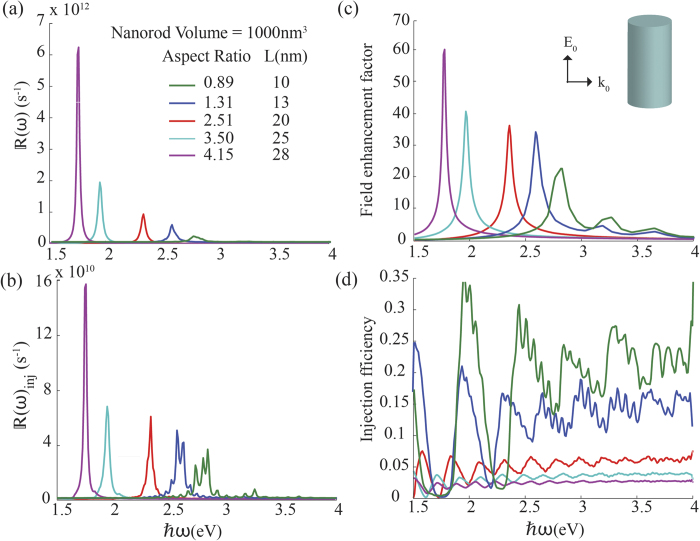
Same as in Fig. 4 except that silver nanorods of different aspect ratios are considered such that they all have the same volume of 1000 nm^3^. For *L* = 10 nm nanorod *τ* was taken to be 300 fs.

Figure 5 shows the effects of varying the aspect ratio of a nanorod while keeping the volume constant. It can be observed that hot-electron generation and injection is highly tunable across the optical spectrum by simply varying the aspect ratio of nanorods. From Fig. 5(a–c) we can say that the spectral profile of hot-electron injection and generation essentially follows the electric field enhancement factor. Further, it can be observed that both 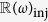 and 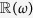 are enhanced while red-shifting with the increasing aspect ratio. This indicates that the number of high energy electrons with the ability to cross over the Schottky barrier increases in nanorods with high aspect ratios. In contrast, Fig. 5(d) shows that the electron injection efficiency declines with increasing aspect ratio, indicating that a higher proportion of excited electrons do not have the capability to cross over to the semiconductor side, even though the overall performance is improving. However since the volume of these nanorods are the same, the injection rate per unit volume is increasing and therefore we can say that nanorods with a higher aspect ratio show better performance in hot-electron harvesting. With the decreasing aspect ratio, the length of the nanorod is reduced as its volume is constant. This results in increased quantization of electron states in the longitudinal direction, which explains the non-smooth, oscillatory behaviour seen in the injection rate curves of nanorods with low aspect ratios.

**Figure 6:**
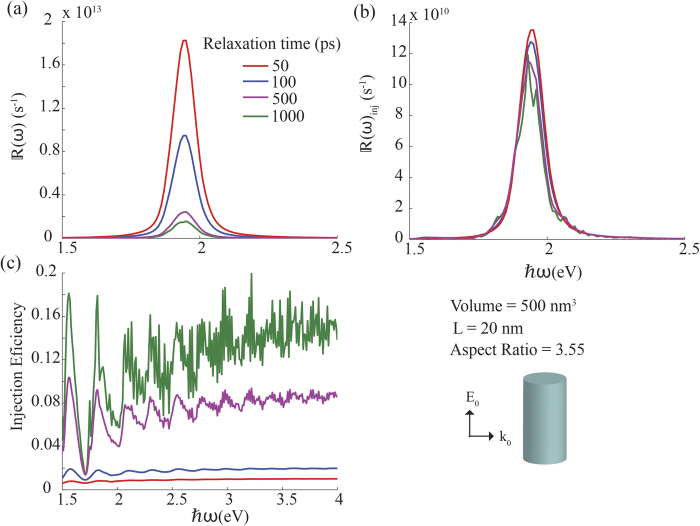
Same as in Fig. 5 except that the electron thermalisation time is varied for silver nanorods of similar volume (500 nm^3^) and the constant aspect ratio (fixed at 3.55).

To recognize the importance of electron’s relaxation time in nanorod hot-electron dynamics, in Fig. 6 we vary *τ* from 50 fs to 1000 fs while keeping all other parameters constant. Figure 6(a,b) suggest that, even though the generation rate is highly influenced by *τ*, the injection rate does not very much with *τ*, reflecting the fact that only the generation of less energetic electrons is affected by *τ*.

**Figure 7:**
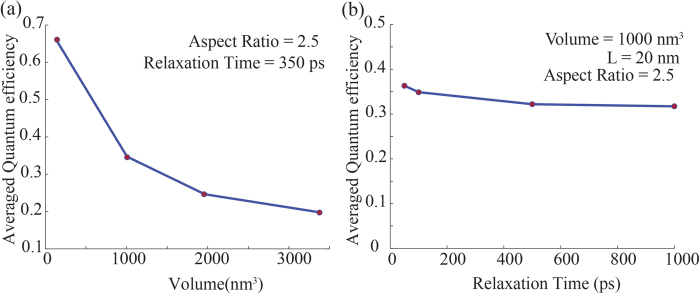
Comparison of averaged quantum efficiency of nanorods over the spectrum 1.5–4(*eV*) with (**a**) varying volume and (**b**) electron thermalization time.

Quantum efficiency of injection is defined as the absorbed photon to injected electron ratio of a nanoparticle. This is a measure of energy conversion efficiency of the system and can be used as a figure of merit for comparison among different energy converting systems. In Fig. 7 we have plotted the averaged quantum efficiency of injection for nanorods of different volumes and thermalisation times using Eqs. (26) and (33) over the frequency range 1.5–4 eV of the solar spectrum. It can be seen that averaged quantum efficiency reduces considerably with increasing volume while it varies little with changing *τ*. These results clearly stress the fact that energy conversion efficiency of nanorods improves with decreasing volume.

We can simplify Eq. (26) for nanorods whose length *L* is long enough that the summation over the quantum number *l*_*i*_ can be replaced with an integral over electron momentum. We introduce the electron momentum in the *z* direction as *k* = *π*(*l*_*i*_ + 1)/*L* and write Eq. (26) as


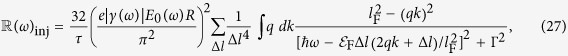


where *q* = *L*/*π*. The integral over the range for momentum *k* can be done analytically using the limits of *l*_*i*_. The final result is


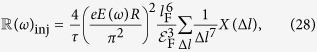



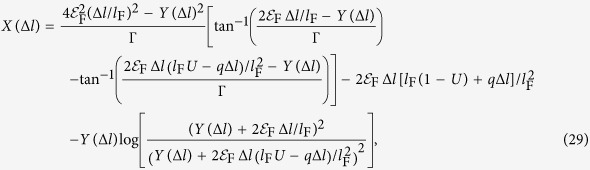


where 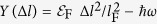. Since 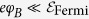, we can use the approximation


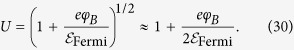


**Figure 8:**
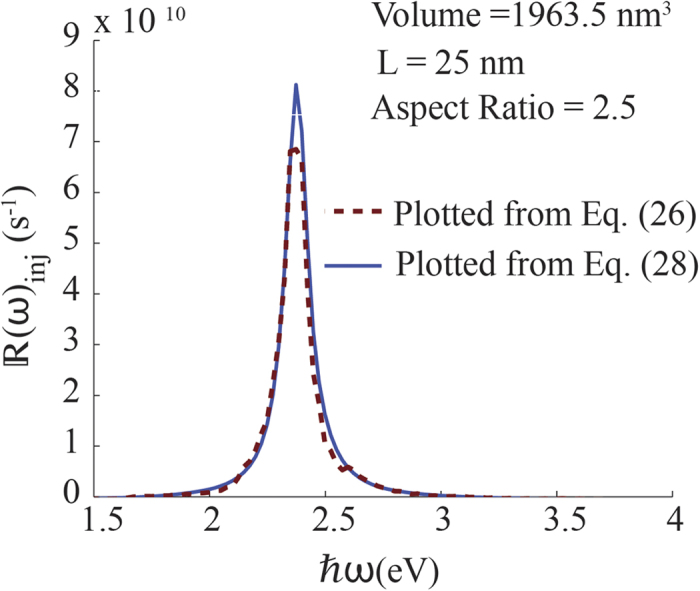
Comparison of electron injection rates calculated from Eq. (26) and Eq. (28).

Equation (28) converges rapidly after few terms of Δ*l*. This tells us that the major contributions to hot-electron injection rate comes from transitions with small differences in the longitudinal quantum number. This is only possible for electrons in states very close to the Fermi level before the plasmon excitation. With a small change in *l*, They can end up with sufficient energy to cross the Schottky barrier. Therefore, electrons close to the Fermi level plays a key role in the injection of hot electrons from nanorod to the semiconductor. In Fig. 8 we have compared the electron injection rates calculated using Eq. (26) and Eq. (28) for a 25 nm-long nanorod. Even though a slight overestimation of the hot carrier injection rate is visible near the peak, we can conclude that Eq. (28) describes the spectrum of hot carriers with sufficient accuracy. It should prove useful in practice because of additional computational effort required for taking the sum over *l*_*i*_ in Eq. (26).

## Discussion

In this article we have derived the rate of high-energy electron generation for a metallic nanorod illuminated optically such that the external electric field is oriented along the nanorod length. We also calculated the rate of injection of these hot electrons from the nanorod to a semiconductor in contact with it using the single-electron wave function and the corresponding eigen energy states.

We note from the energy distribution of the excited electrons, that a high proportion of generated electrons have energies in the vicinity of the Fermi level, which reduces the injection efficiency. Also, major contributions to hot-electron injection comes from electrons that are close to the Fermi level of the metal prior to excitation by plasmons. Using our theory, we show that nanorods have electron injection rates that are several orders of magnitude higher than their spherical or pallet-shaped counterparts, and they should be considered as one of the the most suitable candidates for hot-electron based applications. Also we identify that the frequency of incident light for which the injection rate is the largest and the magnitude of injection rate can be easily tuned by the aspect ratio of the nanorods. In particular, we found that higher aspect ratios result in higher electron injection rates. Further, we found that increasing the volume of the nanorods decreases the injection efficiency per unit volume, when the aspect ratio is constant. We also show that the electron thermalisation time in the metal does not have a significant impact on the electron injection rate. Table 1 summarizes these results.

In our study the injection rate of hot electrons is calculated assuming a smooth interface between the metal and the semiconductor. This assumption imposes the condition that the component of electron’s momentum normal to the interface should be large enough for the electron to cross the Schottky barrier. In practice, imperfections and roughness of this interface can change the direction of momentum of incident electrons and enhance the injection rate.

Localized surface plasmons are collective electron charge oscillations that are excited by light resulting in charge redistributions in metallic nanoparticles. Charge displacement produces corresponding transient dipole moments and a corresponding polarized field. When the incident photon frequency matches the natural frequency of surface electrons oscillating against the restoring force of positive nuclei and boundaries of the particle, the resonance condition is established. The dielectric function defines the polarizability of the electrons inside the particle. It can be calculated by calculating the wave function of the electrons and their eigen states in the material with boundary conditions set by the geometry of the material. Therefore the plasmon resonance is in turn is dependant on the geometry dependant electronic structure/distribution of the particle.

An efficient electron donor mechanism is important to maintain the injection rate during transfer of hot electrons to the semiconductor over sustained periods of time. To minimize the requirement of external wiring on a nano scale, typically electron donors are in the form of a solution in contact with the nanoparticle that causes chemical reactions on the surface. The arrangement we have studied here, a metallic nanorod supported on a semiconductor, allows high degree of contact with the surrounding media, resulting in efficient transfer of electrons from the donor solution to the nanoparticle.

The energetic electrons generated inside the nanoparticle have to reach the metal boundaries before they can be transferred to the semiconductor. These electrons can loose energy through several relaxation processes and may never reach the boundary, especially if the distance they have to travel is larger than the electron’s mean free path in the metal. In contrast, if electrons have high energy, probability of reaching the boundary is higher. Therefore, even though a nanorod with a high aspect ratio can produce large proportion of high energy electrons, if its length is too long compared to electron’s mean free path, most of the electrons may not reach the boundary.

Our work shows that multiple naorods with different aspect ratios can be used to absorb energy over a wide range of the solar spectrum and generate energetic electrons which can be harvested into semiconductors or molecules to create useful work. Our derivations and results provide the much needed theoretical background for understanding such a transfer of plasmon-induced hot electrons using nanorod shaped metals. They should provide guidance in the development and optimization of hot-electron harvesting mechanisms operating over a broad spectral range.

## Methods

### Wave function and electric field enhancement of a nano sphere

Wave function for an electron in a spherical potential well of radius *R*_sph_ is given by, Ψ(*r*, *ϑ*, *φ*)_s_ = *F*(*r*)_*n*,*l*_*Y*_*lm*_(*ϑ*, *φ*), where *r*, *ϑ*, and *φ* are the spherical coordinates and *n*, *l* and *m* are quantum numbers representing a particular electron state. The radial function *F*(*r*)_*n*,*l*_ is the spherical Bessel function of order *l* with its *n*_th_ zero satisfying the boundary conditions of the spherical potential well. The function *Y*_*lm*_(*ϑ*, *ϕ*) represents the spherical harmonics of degree *l* and order *m*. The energy of an electron in this particular state can be written as 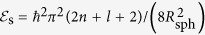. The electric field inside a spherical nano particle can be obtained analytically in the form **E**(*ω*) = 3*ε*_0_/(*ε*_0_ + 2*ε*(*ω*))**E**_0_(*ω*).

### Wave function and electric field enhancement of a nano pallet

The wave function and the energy of a free electron for this geometry are obtaiined using Cartesian coordinates and have the form


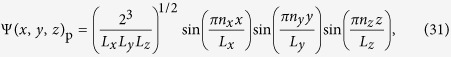



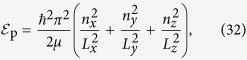


where *L*_*x*_, *L*_*y*_ and *L*_*z*_ are dimensions of the pallet and *n*_*x*_, *n*_*y*_ and *n*_*z*_ are quantum numbers representing the state of the elctron. If the external electric field is assumed to be perpendicular to the pallet, the electric field inside the pallet can be related to the external electric field as **E**(*ω*) = *ε*_0_/*ε*(*ω*)**E**_0_(*ω*).

### Internal quantum efficiency

Internal Quantum efficiency of a nanoparticle can be defined as the ratio between rate of electron injection(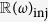) and rate of photon absorption (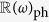):


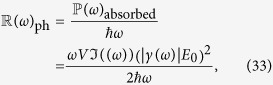


where *V* is the volume of the particle and 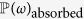 is the power absorbed.

### Material parameters

In all calculations we assume the nanoparticles to be made of silver with a Fermi energy of 5.5 eV. The semiconductor is taken to be TiO_2_, creating a Schottky barrier of 0.4 eV. The complex permittivity of silver is taken from experimental data^46^ and the surrounding medium is assumed to be water with a relative permittivity of 1.8. The illumination intensity is taken as 3.6 × 10^3^ Wcm^−2^.

## Additional Information

**How to cite this article**: Kumarasinghe, C. S. *et al.* Theoretical analysis of hot electron dynamics in nanorods. *Sci. Rep.* **5**, 12140; doi: 10.1038/srep12140 (2015).

## Figures and Tables

**Table 1:** Summery of results obtained for nanorods of various sizes.

**Increasing Parameter**	**Generation rate** 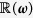	**Injection rate/volume** 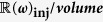	**Injection Efficiency**
Volume of nanorod	Increase	Decrease	Decrease
Aspect Ratio of nanorod	Increase	Increase	Decrease
Electron thermalisation time	Decrease	No significant change	No significant change

## References

[b1] Lee Y. K. *et al.* Surface plasmon-driven hot electron flow probed with metal-semiconductor nanodiodes. Nano letters 11, 4251–4255 (2011). 21916449 10.1021/nl2022459

[b2] Conklin D. *et al.* Exploiting plasmon-induced hot electrons in molecular electronic devices. ACS nano 7, 4479–4486 (2013). 23550717 10.1021/nn401071d

[b3] Mubeen S. *et al.* An autonomous photosynthetic device in which all charge carriers derive from surface plasmons. Nature nanotechnology 8, 247–251 (2013). 10.1038/nnano.2013.18 23435280

[b4] Lee J., Mubeen S., Ji X., Stucky G. D. & Moskovits M. Plasmonic photoanodes for solar water splitting with visible light. Nano letters 12, 5014–5019 (2012). 22916955 10.1021/nl302796f

[b5] Gomes Silva C., Juárez R., Marino T., Molinari R. & Garcia H. Influence of excitation wavelength (uv or visible light) on the photocatalytic activity of titania containing gold nanoparticles for the generation of hydrogen or oxygen from water. Journal of the American Chemical Society 133, 595–602 (2010). 21142160 10.1021/ja1086358

[b6] Mukherjee S. *et al.* Hot electrons do the impossible: plasmon-induced dissociation of h2 on au. Nano letters 13, 240–247 (2012). 23194158 10.1021/nl303940z

[b7] Sil D. *et al.* Seeing is believing: Hot electron based gold nanoplasmonic optical hydrogen sensor. ACS nano 8, 7755–7762 (2014). 25072929 10.1021/nn500765t

[b8] Landy N., Sajuyigbe S., Mock J., Smith D. & Padilla W. Perfect metamaterial absorber. Physical review letters 100, 207402 (2008). 18518577 10.1103/PhysRevLett.100.207402

[b9] Pescaglini A. *et al.* Hot-electron injection in au nanorod-zno nanowire hybrid device for near-infrared photodetection. Nano letters 14, 6202–6209 (2014). 25313827 10.1021/nl5024854

[b10] Akbari A. & Berini P. Schottky contact surface-plasmon detector integrated with an asymmetric metal stripe waveguide. Applied Physics Letters 95, 021104 (2009).

[b11] Giugni A. *et al.* Hot-electron nanoscopy using adiabatic compression of surface plasmons. Nature nanotechnology 8, 845–852 (2013). 10.1038/nnano.2013.207 24141538

[b12] Appavoo K. *et al.* Ultrafast phase transition via catastrophic phonon collapse driven by plasmonic hot-electron injection. Nano letters 14, 1127–1133 (2014). 24484272 10.1021/nl4044828

[b13] Fang Z. *et al.* Plasmon-induced doping of graphene. Acs Nano 6, 10222–10228 (2012). 22998468 10.1021/nn304028b

[b14] Govorov A. O., Zhang H., Demir H. V. & Gunko Y. K. Photogeneration of hot plasmonic electrons with metal nanocrystals: Quantum description and potential applications. Nano Today 9, 85–101 (2014).

[b15] Wijesinghe T., Premaratne M. & Agrawal G. P. Electrically pumped hybrid plasmonic waveguide. Optics express 22, 2681–2694 (2014). 24663560 10.1364/OE.22.002681

[b16] El-Sayed M. A. Small is different: shape-, size-, and composition-dependent properties of some colloidal semiconductor nanocrystals. Accounts of chemical research 37, 326–333 (2004). 15147173 10.1021/ar020204f

[b17] Sikdar D., Rukhlenko I. D., Cheng W. & Premaratne M. Tunable broadband optical responses of substrate-supported metal/dielectric/metal nanospheres. Plasmonics 9, 659–672 (2014).

[b18] Sikdar D., Rukhlenko I. D., Cheng W. & Premaratne M. Unveiling ultrasharp scattering—switching signatures of layered gold—dielectric—gold nanospheres. JOSA B 30, 2066–2074 (2013).

[b19] Wu K., Rodrguez-Córdoba W. E., Yang Y. & Lian T. Plasmon-induced hot electron transfer from the au tip to cds rod in cds-au nanoheterostructures. Nano letters 13, 5255–5263 (2013). 24093501 10.1021/nl402730m

[b20] Fang Z. *et al.* Graphene-antenna sandwich photodetector. Nano letters 12, 3808–3813 (2012). 22703522 10.1021/nl301774e

[b21] Goykhman I., Desiatov B., Khurgin J., Shappir J. & Levy U. Waveguide based compact silicon schottky photodetector with enhanced responsivity in the telecom spectral band. Optics express 20, 28594–28602 (2012). 23263097 10.1364/OE.20.028594

[b22] Hartland G. V. Measurements of the material properties of metal nanoparticles by time-resolved spectroscopy. Physical Chemistry Chemical Physics 6, 5263–5274 (2004).

[b23] Voisin C., Del Fatti N., Christofilos D. & Vallee F. Ultrafast electron dynamics and optical nonlinearities in metal nanoparticles. The Journal of Physical Chemistry B 105, 2264–2280 (2001).

[b24] Inouye H., Tanaka K., Tanahashi I. & Hirao K. Ultrafast dynamics of nonequilibrium electrons in a gold nanoparticle system. Physical Review B 57, 11334 (1998).

[b25] Tian Y., Wang X., Zhang D., Shi X. & Wang S. Effects of electron donors on the performance of plasmon-induced photovoltaic cell. Journal of Photochemistry and Photobiology A: Chemistry 199, 224–229 (2008).

[b26] Govorov A. O., Zhang H. & Gunko Y. K. Theory of photoinjection of hot plasmonic carriers from metal nanostructures into semiconductors and surface molecules. The Journal of Physical Chemistry C 117, 16616–16631 (2013).

[b27] Manjavacas A., Liu J. G., Kulkarni V. & Nordlander P. Plasmon-induced hot carriers in metallic nanoparticles. ACS nano 8, 7630–7638 (2014). 24960573 10.1021/nn502445f

[b28] Langhammer C., Yuan Z., Zoric I. & Kasemo B. Plasmonic properties of supported pt and pd nanostructures. Nano letters 6, 833–838 (2006). 16608293 10.1021/nl060219x

[b29] Fowler R. H. The analysis of photoelectric sensitivity curves for clean metals at various temperatures. Physical Review 38, 45 (1931).

[b30] Knight M. W. *et al.* Embedding plasmonic nanostructure diodes enhances hot electron emission. Nano letters 13, 1687–1692 (2013). 23452192 10.1021/nl400196z

[b31] Frese K. W. & Chen C. Theoretical models of hot carrier effects at metal-semiconductor electrodes. Journal of The Electrochemical Society 139, 3234–3243 (1992).

[b32] Dowgiallo A.-M., Schwartzberg A. M. & Knappenberger K. L. Jr Structure-dependent coherent acoustic vibrations of hollow gold nanospheres. Nano letters 11, 3258–3262 (2011). 21714493 10.1021/nl201557s

[b33] Ungureanu C., Rayavarapu R. G., Manohar S. & van Leeuwen T. G. Discrete dipole approximation simulations of gold nanorod optical properties: Choice of input parameters and comparison with experiment. Journal of Applied Physics 105, 102032 (2009).

[b34] Averitt R. D., Westcott S. L. & Halas N. J. Linear optical properties of gold nanoshells. JOSA B 16, 1824–1832 (1999).

[b35] Venermo J. & Sihvola A. Dielectric polarizability of circular cylinder. Journal of electrostatics 63, 101–117 (2005).

[b36] DePrince A. E. & Hinde R. J. Accurate computation of electric field enhancement factors for metallic nanoparticles using the discrete dipole approximation. Nanoscale research letters 5, 592–596 (2010). 20672062 10.1007/s11671-009-9511-7 PMC2894168

[b37] Grynberg G., Aspect A. & Fabre C. Introduction to quantum optics: from the semi-classical approach to quantized light (Cambridge university press, 2010).

[b38] Link S. & El-Sayed M. A. Spectral properties and relaxation dynamics of surface plasmon electronic oscillations in gold and silver nanodots and nanorods. The Journal of Physical Chemistry B 103, 8410–8426 (1999).

[b39] Lermé J. *et al.* Effects of confinement on the electron and lattice dynamics in metal nanoparticles. The European Physical Journal D-Atomic, Molecular, Optical and Plasma Physics 34, 199–204 (2005).

[b40] Voisin C. *et al.* Size-dependent electron-electron interactions in metal nanoparticles. Physical review letters 85, 2200 (2000). 10970497 10.1103/PhysRevLett.85.2200

[b41] Groeneveld R. H., Sprik R. & Lagendijk A. Femtosecond spectroscopy of electron-electron and electron-phonon energy relaxation in ag and au. Physical Review B 51, 11433 (1995). 10.1103/physrevb.51.11433 9977873

[b42] Bigot J.-Y., Halté V., Merle J.-C. & Daunois A. Electron dynamics in metallic nanoparticles. Chemical Physics 251, 181–203 (2000).

[b43] Gueymard C., Myers D. & Emery K. Proposed reference irradiance spectra for solar energy systems testing. Solar energy 73, 443–467 (2002).

[b44] Tabak J. Solar and geothermal energy (Infobase Publishing, 2009).

[b45] Dixon A. E. & Leslie J. D. Solar Energy Conversion: An Introductory Course (Elsevier, 2013).

[b46] Johnson P. B. & Christy R.-W. Optical constants of the noble metals. Physical Review B 6, 4370 (1972).

